# Pedunculated Polypoidal Anal Canal Lymphangioma in an Adult Male: A Report of a Rare Case and Review of the Literature

**DOI:** 10.7759/cureus.105869

**Published:** 2026-03-26

**Authors:** Keerthanah Thiagarajan, Jyoti Verma, Jyotsna N Bharti, Marthandam Srikanth

**Affiliations:** 1 Pathology and Laboratory Medicine, All India Institute of Medical Sciences, Mangalagiri, Mangalagiri, IND; 2 General Surgery, All India Institute of Medical Sciences, Mangalagiri, Mangalagiri, IND

**Keywords:** adult, anal canal, lymphangioma, male, pedunculated

## Abstract

Lymphangiomas are benign malformations of the lymphatic system and are often misdiagnosed due to relatively vague and nonspecific symptoms. They most commonly involve the mucous membranes and skin of the head and neck region in pediatric patients. Gastrointestinal tract and anal canal involvement are exceedingly rare, with very limited cases reported in the literature. An adult male in his early twenties presented with complaints of a bloating sensation in his abdomen. Routine investigations were performed. Colonoscopy revealed a solitary, small, pedunculated, polypoid lesion at the 9 o’clock position. Complete excision of the lesion was performed and sent for histopathological examination (HPE), which showed numerous thin-walled, dilated, multicystic cavernous lymphatic channels. On immunohistochemical examination (IHC), the spaces were lined by flattened endothelial cells stained by D2-40 and CD31. Anal canal lymphangiomas are rare, especially in adults. Previously reported cases involved patients aged 32-69 years, making our case among the youngest described, in their early twenties. Unlike common presentations of rectal bleeding or discomfort, our patient reported a bloating sensation. Colonoscopy revealed a polypoidal lesion at the 9 o’clock position of the anal canal. The diagnosis of anal lymphangioma was established and confirmed by IHC with D2-40 and CD31. This case represents the spectrum of age and symptoms of anal lymphangiomas and highlights the need to consider them as an important differential in young adults when dealing with anal canal polyps. Complete excision remains curative with excellent outcomes. This case underscores the need to consider lymphangioma in the differential diagnosis of perianal masses and highlights the pivotal role of histopathology along with immunohistochemical analysis in establishing an accurate diagnosis, which is necessary for formulating appropriate management.

## Introduction

Lymphangiomas are benign, non-neoplastic malformations of the lymphatic system, most commonly arising during early childhood. They predominantly involve the cervicofacial and axillary regions, while gastrointestinal manifestations are infrequent [1]. In particular, primary anal canal lymphangiomas are exceedingly rare [2]. We identified only seven publications that adequately described such cases. Because they are found in an unusual part of the body and do not show serious symptoms, they are often mistaken for more common or possibly cancerous anorectal conditions [2-4]. Recognizing this rare entity is crucial to avoid diagnostic pitfalls and to facilitate accurate histopathological evaluation and appropriate clinical management. Fewer than ten cases, only seven to date, have been reported in the literature [2,5], and our case adds to this limited body of evidence. To the best of our knowledge, our scenario marks the first documented case of anal canal lymphangioma from India.

## Case presentation

**Figure 1:**
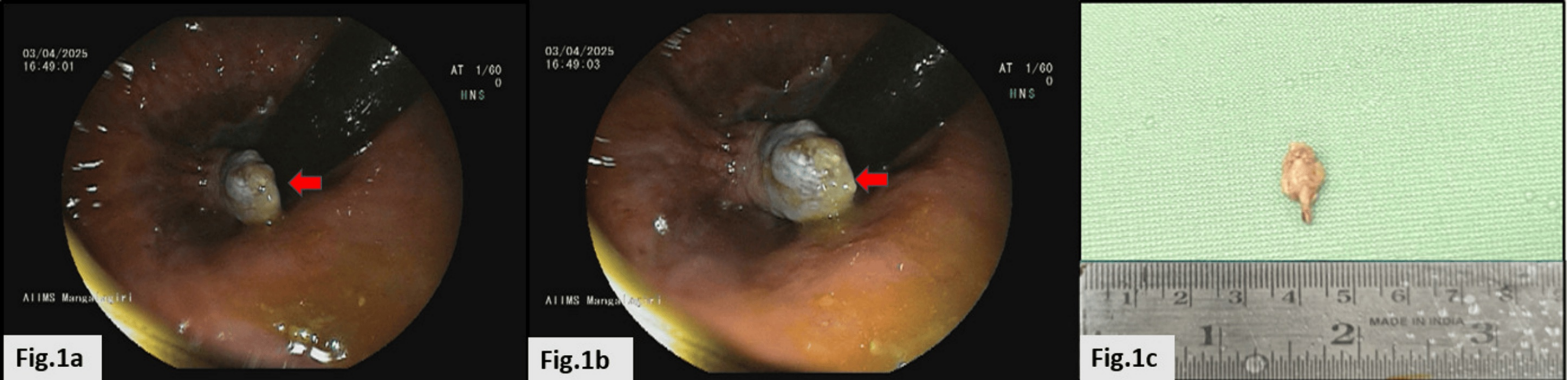
Colonoscopic examination and gross received at the department of pathology (a and b) Solitary anal mucosal polyp at the 9 o’clock position. (c) Single tiny polypoidal soft tissue fragment.

An adult male in his early twenties presented to the outpatient department of general surgery with complaints of a bloating sensation post-meal, which was relieved upon defecation and occasionally associated with blood-stained stools. There was no history of rectal discharge, anorectal trauma, or prior anorectal surgery. Differentials of anal lesions such as external hemorrhoids, anal tags, and benign rectal polyps were considered. A colonoscopy examination revealed a solitary, soft, polypoidal lesion at the 9 o’clock position of the anal canal (Figure 1a-b). On routine investigations, hematological and biochemical parameters were within normal limits. Surgery was performed, and the specimen was sent for gross examination. It was a single, tiny polypoidal soft-tissue fragment measuring 0.8 × 0.7 × 0.4 cm, with a stalk measuring 0.2 cm (Figure 1c).

**Figure 2:**
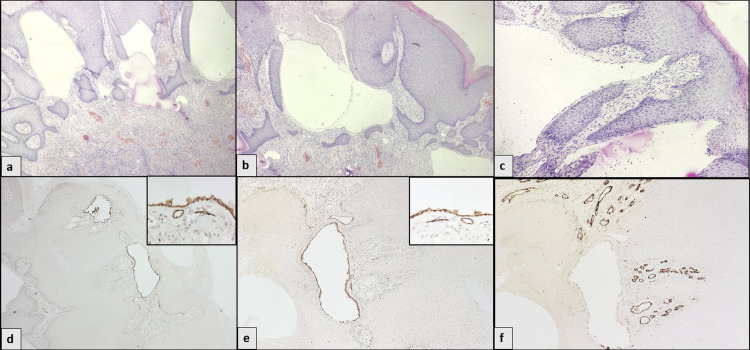
Histopathology images and immunohistochemistry images for D2-40, CD31, and CD34 (a-c) Numerous thin-walled, dilated, multi-cystic cavernous lymphatic channels containing homogenous, faint, eosinophilic secretions (HE; x40; x100, x200). (d-f) IHC staining for D2-40 (x100, x400 (inset)), CD31 (x100, x400 (inset), in endothelial lining of lymphatics), and CD34 (x100) in endothelial lining of vessels. IHC: immunohistochemical examination.

Upon histopathological examination (HPE), hyperplastic anoderm lined the polypoidal tissue, displaying hyperkeratosis, irregular acanthosis, and elongated and focal anastomosing rete ridges. There was superficial melanin incontinence. It comprised numerous thin-walled, dilated, multicystic cavernous lymphatic channels containing homogeneous, faint, eosinophilic secretions. These lymphatic channels were lined by flattened endothelial cells, extended superficially, and were located near the pedicle of the polyp. The stroma comprised chronic inflammatory infiltrate and intervening dilated and congested blood vessels. No evidence of mitosis, dysplasia, or malignancy was identified (Figure 2a-c). On immunohistochemical examination (IHC), the flattened endothelial cells lining the spaces were highlighted by D2-40 and CD31, thus confirming the lymphatic endothelial origin. CD34 positivity was noted in the interspersed dilated blood vessels (Figure 2df). The entities that may enter the differentials and pose diagnostic challenges were external hemorrhoids, skin tags, condyloma acuminatum, hemangioma, soft tissue tumors, anal canal polyps, and carcinomas. HPE and IHC helped in establishing the final diagnosis. The patient was admitted, the lesion was excised, and the patient was discharged the next day. At the one-month follow-up, the surgical site had healed completely. While excision of a 0.8 cm polyp under local anesthesia is usually an outpatient procedure, the patient was admitted owing to logistical considerations at our autonomous government institution, which manages a high volume of patients. Additionally, hospitalization was required for bowel preparation prior to colonoscopy. No peri-procedural or post-procedural complications occurred, and the duration of stay was unrelated to the surgical procedure itself. There were no signs of inflammation or complication at that site. The patient remained asymptomatic thereafter. Currently, after six months, the patient is doing well. A longer follow-up period is required to understand the recurrence potential of the lesion.

## Discussion

Anal canal lymphangiomas are uncommon, benign malformations of the lymphatic system, accounting for approximately 4% of all vascular tumors. They most frequently present in the cervicofacial and axillary regions. The anal canal is an uncommon site for lymphangiomas, with fewer than ten cases documented in the literature, as illustrated in Table 1 [1].

**Table 1:** All the cases of anal canal lymphangioma reported in the literature so far (year-wise) IHC: immunohistochemical examination.

Year	Age/Gender	Clinical Presentation	Radiology/Endoscopy	Gross Findings	Microscopic Findings	IHC
Thomas et al., 2024 [6]	47/F	Anal mass, bleeding	Endoscopy: Submucosal lesion	Polypoid lesion	Dilated lymphatic channels in submucosa	NA
Ismaeel et al., 2024 [5]	32/M	Perianal swelling	Solitary, soft, pedunculated polyp, paler than the surrounding mucosa	Polypoid lesion	Cystically dilated lymphatic vessels	D2-40+
Chishti et al., 2021 [7]	55/M	Rectal bleeding	Endoscopy: Smooth submucosal lesion	Soft, polypoid mass	Dilated lymphatic vessels with thin walls	D2-40+, CD31+
Hussain et al., 2020 [8]	69/F	Rectal bleeding	Anal canal polyp	Polypoid lesion	Dilated lymphatic spaces in the submucosa	Not done
Karapashis et al., 2018 [3]	42/M	Bleeding per rectum	Colonoscopy: Submucosal lesion in the anal canal	Polypoidal, soft lesion	Numerous lymphatic vessels in the stroma	D2-40+, CD31+
Mannami et al., 2014 [2]	42/F	Rectal bleeding	Endoscopy: Semitranslucent, bluish polyp	Not mentioned	Dilated lymphatic spaces in the lamina propria	CD34+ in endothelial cells of vessels
Val Bernal et al., 2008 [4]	40/F	Rectal bleeding, anal discomfort	An ulcerated, soft lesion	Pedunculated polypoid mass	Cavernous lymphangioma: Dilated lymphatic channels lined by endothelium	D2-40+, CD31+
Index case	24/M	Bleeding per rectum, anal polyp	Colonoscopy: Smooth, pinkish submucosal lesion	Excised polypoidal lesion	Dilated lymphatic channels in the submucosa, lined by bland endothelial cells	D2-40+, CD31+, CD34+ in vessel endothelium

The clinical presentation of anal canal lymphangiomas is often subtle and nonspecific, contributing to frequent misdiagnosis. These lesions may mimic more common anorectal conditions such as hemorrhoids, skin tags, or polyps. Symptoms typically include rectal bleeding, abdominal discomfort, or may even be asymptomatic, as highlighted in cases by Thomas et al. and Chishti et al. [6,7]. The present case of an individual in his early twenties with postprandial bloating and intermittent rectal bleeding aligns with this variable symptomatology. Bloating was a nonspecific associated symptom reported by the patient and was not attributed solely to the anal canal lesion. Notably, our case is one of the youngest adult patients reported, contrasting with the age spectrum observed in previously documented cases, which ranged from 32 to 69 years [3,5,8].

A comparison of clinical features reveals that while rectal bleeding was a common presenting symptom in most cases, the sensation of postprandial bloating described by our patient has not been widely reported, thus adding a novel clinical detail to the spectrum. In terms of gender, our case contributes to the slight male predominance observed in previous reports, with a male-to-female ratio of approximately 5:3 [3,4,6].

Endoscopic findings in previously reported cases describe it as a polypoidal, soft, and often translucent lesion. Mannami et al. provided the first endoscopic description using narrow-band imaging, identifying a 20 mm bullous lesion with a clear, compressible appearance [2]. Our case revealed a much smaller (0.8 cm) lesion during colonoscopy, described as solitary and pedunculated, reflecting the size variability reported in the literature (0.8 cm to 2 cm) [3,4]. It appeared as a sessile polyp. The benign appearance and nonspecific clinical presentation can result in under-recognition unless histopathological examination is performed.

Surgically, complete excision remains the preferred treatment modality. Our case, excised under local anesthesia without colonoscopic guidance, showed no recurrence at the follow-up duration of one year. On digital per rectal examination, the polyp was palpable with the index finger. Bipolar cautery was used. The lesion was excised and left open. These findings are similar to those reported by Val-Bernal et al. and Karapashis et al., supporting that careful surgical removal is usually sufficient to cure most cases.

Histopathologically, anal canal lymphangiomas are predominantly of the cavernous subtype, characterized by multiple dilated lymphatic spaces lined by flattened endothelial cells. The lesion often shows a fibroinflammatory stroma and lacks features of dysplasia or malignancy. The index case showed hyperplastic anoderm with hyperkeratosis and dilated lymphatic channels, consistent with findings by Karapashis et al. and Val-Bernal et al., who also described dilated lymphatic vessels in a pedunculated configuration [3,4].

IHC for lymphatic endothelial markers (D2-40 and CD31) is consistently expressed across cases, with CD34 highlighting associated blood vessels. Our findings mirrored this typical profile, confirming the diagnosis. These IHC results were similar to those in the cases reported by Thomas et al., Chishti et al., and Ismaeel et al., indicating a strong diagnostic utility for this panel [5-7].

Despite the overall benign nature, proper recognition of anal canal lymphangiomas is critical. Their infrequency and clinical similarity to prevalent anorectal lesions require histopathological and immunohistochemical validation for precise diagnosis. Our case, to the best of our knowledge, is the first reported case from India, contributing valuable insights to the limited global data and emphasizing the need for increased awareness of this entity among clinicians and pathologists.

## Conclusions

Lymphangioma should be considered in the differential diagnosis of anal canal lesions. Histopathological and immunohistochemical analyses are essential for establishing an accurate diagnosis. Complete surgical excision is typically curative and associated with a low risk of recurrence. The rarity of adult anal canal lymphangiomas suggests the possibility of underdiagnosis or underreporting, highlighting the importance of increased clinical awareness and more comprehensive documentation.
